# Feasibility of Monte-Carlo algorithm in comparison with collapse-cone dose calculation algorithm of a commercial treatment planning system in the presence of high-density metallic implant: a dosimetric study

**DOI:** 10.1186/s43046-020-00057-x

**Published:** 2021-01-07

**Authors:** Manindra Bhushan, Deepak Tripathi, Girigesh Yadav, Lalit Kumar, Rahul Lal Chowdhary, Anjali K. Pahuja, Tamilarasu Suresh, Sushil Kumar Shukla, Swarupa Mitra

**Affiliations:** 1 Division of Medical Physics & Department of Radiation Oncology, Rajiv Gandhi Cancer Institute and Research Centre, Sector-5, Rohini, New Delhi, 110085 India; 2 Amity School of Applied Sciences, Amity University (AUUP), Noida, India; 3 Dr. APJ Abdul Kalam Technical University, Lucknow, UP India

**Keywords:** Implant, Collapse-cone-convolution, Box technique, Monte-Carlo algorithm, Carcinoma cervix

## Abstract

**Background:**

The number of people with implanted hip prosthesis has grown worldwide. For radiotherapy planning of patients with hip implants, few main challenges are encountered. The aim of the present study was to evaluate the feasibility of different planning algorithms in the presence of high-density metallic implant in the treatment of patients with carcinoma cervix.

**Results:**

It was found that *D*_98%_ were 44.49 ± 0.11, 44.51 ± 0.13, 44.39 ± 0.22, and 44.45 ± 0.16 Gy for 4FMC6MV (4-field technique calculated with Monte-Carlo algorithm and 6 MV photon energy), 4FMC6MV_WP (4-field technique calculated with Monte-Carlo algorithm and 6 MV photon energy without prosthesis), 4FCC6MV (4-field technique calculated with collapse-cone-convolution algorithm and 6 MV photon energy), and 4FCC6MV_WP (4-field technique calculated with collapse-cone-convolution algorithm and 6 MV photon energy without prosthesis) respectively. Similarly, *D*_2%_ were 49.40 ± 0.84, 49.05 ± 0.76, 48.97 ± 0.91, and 48.57 ± 0.85 Gray (Gy) for 4FMC6MV, 4FMC6MV_WP, 4FCC6MV, and 4FCC6MV_WP respectively. The present study has not suggested any major difference between the Monte-Carlo (MC) and collapse-cone-convolution (CCC) calculation algorithm in the presence of high-Z metallic implants. Volume of bowel receiving 15 Gy dose has shown a significant difference with prosthesis cases. This study investigates that hip prosthesis creates considerable changes in the treatment planning of cervical malignancies.

**Conclusion:**

CCC algorithm is in good agreement with MC calculation algorithm in the presence of high-density metallic implants in terms of target coverage and avoidance organ sparing except few parameters.

## Background

Patients undergoing pelvic radiation therapy account for a significant proportion of all patients undergoing radiation therapy. The number of people with implanted hip prosthesis has grown worldwide over the past several decades. According to report of American Association of Physicist in Medicine Task Group No. 63 [1], 1–4% of patients with radiation therapy have prosthesis. These devices are typically made from high atomic number (high-Z) elements (high-Z materials are classified as a material with an atomic number higher than the cortical bone). Hip prostheses are made from cobalt-chrome-molybdenum (Co-Cr-Mo) alloy because it is believed to be the strongest balance of mechanical strength and corrosion resistance [2]. However, both stainless steel and titanium hip prostheses are also available for clinical use.

For radiation therapy treatment planning of patients with hip implants, two main challenges are encountered. First, the hip implant made from a high-Z material produces significant artifacts in the computed tomography (CT) images that have a vital role in radiation therapy dose calculation. Second, the presence of high-Z material produces a considerable attenuation to the beam down range of the implant, which alters the dose distribution in that area.

In three-dimensional (3D) treatment planning, tomographic images are used. Although CT is able to provide complete information about the densities of the internal structures and their geometries in most clinical conditions, high-density implants produce artifacts and hence the beam-hardening artifacts and CT number errors and produce partial image loss and geometry error in the CT images. These artifacts cause considerable dose calculation errors in computerized treatment planning systems (TPSs).

There are numerous studies, which have been carried out in the field for comparison of the results of Monte-Carlo (MC) measurement with the calculations commercial TPSs on dose perturbation produced by prostheses in radiation therapy. Some of these studies are reviewed here as follows.

Catli and Tanir [3] have evaluated the performance of Eclipse TPS on dose distribution in the presence of hip prostheses. They showed that when a high-Z material is applied in implant, alteration of dose can exist due to the radiation scattering from the prosthesis. The variance in dose observed in the study depended on material type, atomic number, and density as well as photon energy. The dose perturbation caused by hip implants was considerable and could not be predicted precisely by the pencil beam convolution (PBC) method. The results showed that a Monte-Carlo (MC) algorithm-based planning system should be used for accurate dose calculation for the patients with hip prostheses.

Keall et al. [4] investigated the capability of different dose calculation algorithms to obtain accurate treatment plans in the case of high-Z implants. In their study, dose distributions using MC, pencil beam convolution (PBC), superposition, and heterogeneity correction algorithms in phantom and patient geometries with high-Z prostheses were calculated. The results indicated that treatment plans have similar isodose curves, tumor control probability, normal tissue complication probability, and dose-volume histograms as the MC plan which was calculated with superposition algorithm. On the other hand, treatment plans calculated with either no heterogeneity correction or PB methods varied significantly from the MC plans.

A common method to minimize the scattered dose due to hip prosthesis is to avoid the same in the treatment planning. There are chances of the beam exiting through the prosthesis most of the times. There are treatment planning systems available which can estimate the dose in the vicinity of high-density prosthesis implant with improved accuracy but still cannot predict the doses accurately in the interface regions.

The aim of the present study was to evaluate the feasibility of different planning algorithms in the presence of high-density metallic implant in the treatment of patients with carcinoma cervix.

## Methods

### Patient selection

Twenty patients of diagnosed cases of carcinoma cervix with and without high-Z implant each were taken up for the study. The patients were selected in such a way that all were having prosthesis implant in their right-side femoral bone. The material of prosthesis for the selected patients was “titanium” (composition: carbon 0.08%, oxygen 0.13%, iron 0.25%, aluminum 5.6–6.5%, vanadium 3.5–4.5%, and titanium 88.5–91.0%; average electron density 3.74 relative to water; diameter of femoral heads of prosthesis ranging from 40 to 54 mm). All these information are required for executing the dose computations with precision. Bhushan et al. [5] reported that dose perturbation changes with a factor for different energies also. Patients had already completed the total course of treatment. Plans were re-optimized and calculated for collapse-cone-convolution (CCC) algorithm.

### Simulation and target delineation

Patients were taken for mold room procedure and thermoplastic cast were made for immobilization. Radio-opaque fiducial markers were used on pelvic region at the level of pubic symphysis to set the reference position. Computed tomographic (CT) scans were acquired with slice thickness of 3 mm. We have used Siemens’s CT unit (model: Somatom Sensation Open) of our radiotherapy department for the above procedure. Full bladder protocol was followed for all the patients for simulation as well as for the whole course of treatment. Scans were acquired from the level of L2 vertebra to 5 cm below the ischial tuberosity. These scans were transferred in DICOM (Digital Imaging and Communication in Medicine) format to the contouring workstations. MONACOSIM (Contouring station; Elekta Medical Solutions) was used for delineation of planning target volume (PTV) and other critical structures by a qualified radiation oncologist.

**Fig. 1:**
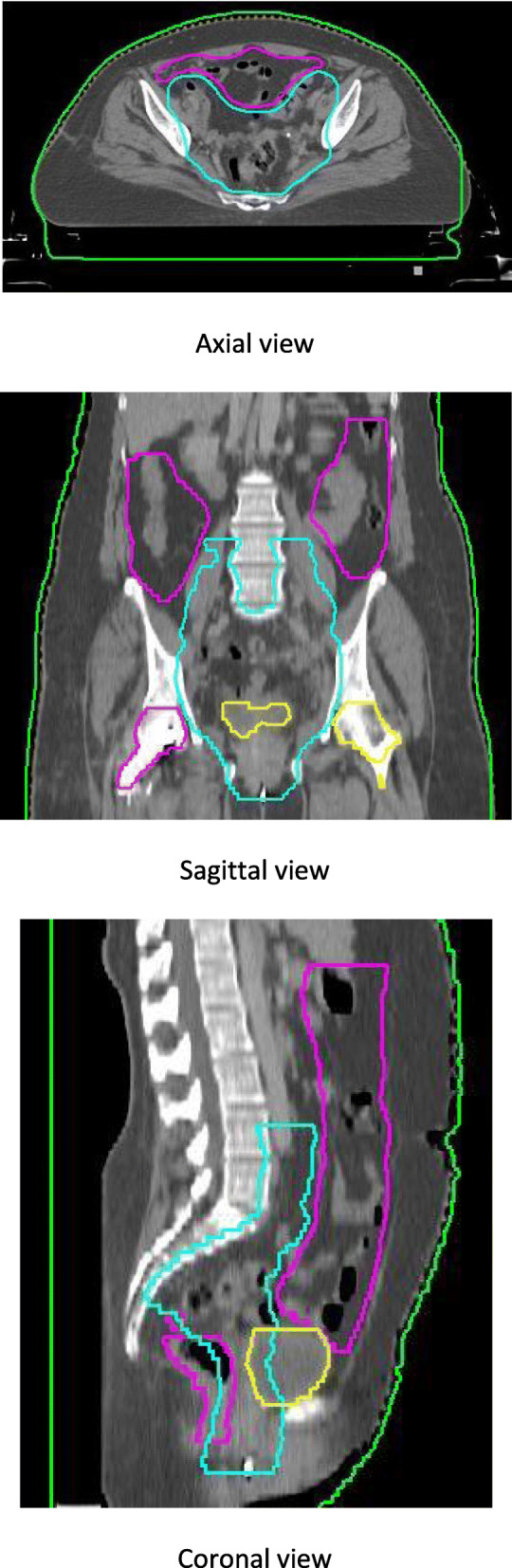
Delineation of contours showing prosthesis in the right side

Clinical target volume (CTV) included the lymphonodal regions (presacral, obturator, and iliac region), uterus, adnexa, and vagina. A margin of 5 mm was given to CTV to generate PTV. The organs at risk (OARs) delineated were the bladder, rectum, small bowel, and femoral heads. Bladder was contoured from apex to dome while the rectum was delineated from the anorectal junction, defined by where the levator muscles fuse with the external sphincter muscles, to the recto-sigmoid junction. Bilateral femoral heads were contoured along with the proximal femur inferiorly from the lowest level of ischial tuberosity and superiorly to the top of the ball of the femur including the trochanters as shown in Fig. 1 [6].

### Treatment planning

Isocentric plans were generated using MONACO (Elekta Medical Solutions) treatment planning system (TPS). Plans were made for a prescribed dose of 45 Gy in 25 fractions with a dose of 1.8 Gy per fraction. Planner medical physicist used the gantry angles as 0°, 180°, 90°, and 270° with collimator and couch angle 0°. Beam arrangement was kept identical in all the plans to analyze the effect of calculation algorithms. MONACO facilitates the planner to use different calculation algorithms like collapse-cone-convolution (CCC) and Monte-Carlo (MC) for plan calculation. Our planning goal was to achieve 98% prescription dose to 100% of PTV volume, i.e., the PTV should be covered with at least 98% of prescribed dose. The nearby critical structures were kept as low as possible and the “hot spots, i.e., dose more than 110% of prescribed dose” were allowed within the PTV only.

### Calculation algorithms (pencil beam convolution, collapse-cone-convolution, and Monte-Carlo)

Pencil beam convolution (PBC) calculation depends on energy fluence and incorporates head scatter modeling. It depends on a two-dimensional pencil beam convolution for volume reconciliation. Inhomogeneities are taken care of by a proportional way length amendment for the essential portion commitment and a one-dimensional convolution along fan lines with an exponential for dispersed radiation.

Collapse-cone convolution (CCC) calculation algorithm is a methodology wherein a ray-trace procedure through the irradiated object is used to get the TERMA (total energy released per unit mass) at all points in the dose calculation matrix. The TERMA is divided into an essential part (collision kerma) and a scatter part.

Monte-Carlo (MC) calculation from Elekta (MONACO/MC) depends on a fluence model utilizing virtual energy fluence (VEF) model, while the dose distribution inside the patient is determined by the photon voxel MC calculation (XVMC).

### Evaluation parameters

PTV was evaluated for *D*_98%_ (dose received by 98% volume of PTV), *D*_2%_ (dose received by 2% volume of PTV), *D*_50%_ (dose received by 50% volume of PTV), *D*_max_ (maximum dose received by PTV), *D*_mean_ (average dose received by PTV), *V*_107%_ (percentage volume of PTV receiving 107% of prescription dose), and *V*_110%_ (percentage volume of PTV receiving 110% of prescription dose).

Bladder and rectum were evaluated for *D*_max_ (maximum dose received by respective organ), *D*_mean_ (mean dose received by respective organ), *D*_2cc_ (dose received by 2 cc volume of respective organ), *V*_45Gy_ (percentage volume of respective organ receiving 45Gy), and *V*_50Gy_ (percentage volume of respective organ receiving 50Gy).

Parameters evaluated for bowel were *V*_5Gy_ (percentage volume of particular organ receiving 5Gy), *V*_15Gy_ (percentage volume of particular organ receiving 15Gy), *V*_30Gy_ (percentage volume of particular organ receiving 30Gy), and *D*_mean_ (mean dose received by particular organ). Mean dose to femoral heads and total monitor units (TMU) were also evaluated.

### Dose-volume histogram (differential and cumulative)

Dose-volume histograms (DVH) are the best method to modify treatment procedures to diminish the intense and long term radiation related toxicities. There are two forms of dose-volume histograms categorized as differential DVH and cumulative DVH.

Differential DVH is a plot of volume receiving a dose inside a predetermined dose interval (or dose bin) as a function of dose. A differential DVH shows dose variation inside a given structure. A differential DVH resembles a typical histogram. The structure volumes are given on the vertical axis, and the bin doses are given on the horizontal axis. The height of each bar of a differential DVH shows the volume of structure accepting a dose given by the bin.

Cumulative DVH is a plot of the volume of a given structure accepting a specific dose or higher as a function of dose. The volume is given on the vertical axis, and the bin doses are on the horizontal axis similarly as the differential DVH, in any case, the height of each bar represents the volume of structure accepting more noteworthy than or equivalent to that dose. Since 100% of the volume consistently gets in any event zero portion, all DVHs start at 100% on the vertical axis. With very small bin sizes, cumulative DVHs resemble a smooth line diagram. The cumulative DVH is utilized most normally in radiation treatment. Cumulative DVHs generally incorporate the target volume and nearby critical structures to the treatment volume.

Despite the fact that DVHs are significant for assessment of dose distribution, they offer no spatial information and do not show where inside a structure the dose is delivered. Dose-volume histograms are ought to be utilized related to 3D dose distribution to see where the dose is actually received.

### Conformity indices

The conformity index is a tool which essentially evaluates the level of consistency between isodoses, tumor shapes, and organs at risk contours by geometric intersection techniques. An analysis of conformity indices for isodoses more prominent than the 95% isodose would give data about the level of homogeneity of irradiation of the target. Conformity index (CI) was calculated using following formulae:

Conformity number (CN) [7]: (TV_RI_/TV)*(TV_RI_/*V*_RI_)

where TV_RI_: target volume covered by the reference isodose (98%)

TV: target volume

*V*_RI_: volume of reference isodose, i.e., 98%

Conformity index RTOG (CI_RTOG_) [8]: V_RI_/TV

Healthy tissue conformity index (CI_HT_) [9]: TV_RI_/V_RI_

Conformity number (CN) incorporates the fraction of target inclusion and the portion of volume of healthy tissues receiving a dose more prominent or equivalent to the prescription dose. The CN index ranges from 0 to 1. The estimation of CI_RTOG_ equivalent to 1 compares to perfect adaptation. If there should be an occurrence of significant worth more prominent than 1 shows the consideration of solid tissues in the irradiated volume. CI_HT_ measures the extent of the volume of the reference isodose containing the objective volume for example in a roundabout way the volume of healthy tissue remembered for the reference isodose. It ranges from 0 to 1 (from “no spatial concordance” to “flawless compliance”). As indicated by Lomax and Scheib, irradiation is considered as conformal if and only if this index is equivalent to or more prominent than 0.6.

### Homogeneity indices

HI_ICRU_: (*D*_2_ − *D*_98_)/*D*_50_ [10, 11]

HI: *D*_2_/*D*_98_ [12]

HI_RTOG_ : *I*_max_/RI [8]

where *I*_max_: maximum isodose in the target

RI: reference isodose

HI_ICRU_ was mentioned in ICRU Report No. 83 and was defined by the ratio of difference of *D*_2%_ and *D*_98%_ and dose received by 50% of PTV. A value of HI close to 0 is considered as homogeneous plan. As per the literature published by Semenenko et al. [12], the ideal value of HI is 1, and it increases as the plan becomes less homogeneous. As per recommendations of RTOG, if the “homogeneity index” (HI_RTOG_) is less or equal to 2, treatment is considered to comply with the protocol. If the index varies between 2 and 2.5, the protocol is considered to be minorly violated, but when the index exceeds 2.5, the protocol is considered as major violation and not acceptable.

### Data and statistical analysis

DVH was plotted by the TPS for target volumes and different organs at risk. The subjective examination of target coverage was finished utilizing axial, sagittal, and coronal slices. For factual examination, the two-tailed paired *t* test was performed to compare the results with the statistical significance level set at *P* ≤ 0.05, between with and without prosthesis plans. Data analysis was performed by the Statistical Package of Social Sciences software (SPSS version 23.0), designed in Chicago, USA.

## Results

### Planning target volumes (PTV)

**Fig. 2:**
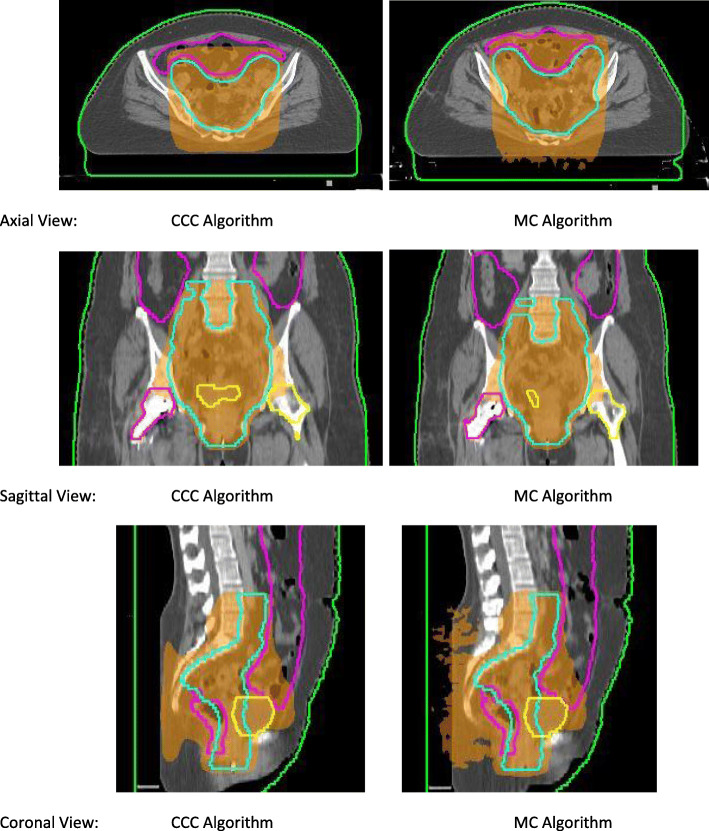
Dose coverage of 95% of prescription dose to PTV

**Fig. 3:**
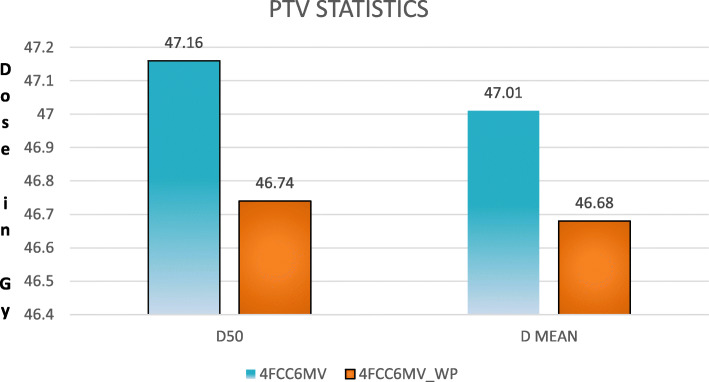
PTV dose comparison for D_50_ and *D*_mean_ using CCC algorithm

Plans were evaluated and found that *D*_98%_ were 44.49 ± 0.11, 44.51 ± 0.13, 44.39 ± 0.22, and 44.45 ± 0.16 Gy for 4FMC6MV (4-field technique calculated with Monte-Carlo algorithm and 6 MV photon energy), 4FMC6MV_WP (4-field technique calculated with Monte-Carlo algorithm and 6 MV photon energy without prosthesis), 4FCC6MV (4-field technique calculated with Collapse-Cone-Convolution algorithm and 6 MV photon energy), and 4FCC6MV_WP (4-field technique calculated with collapse-cone-convolution algorithm and 6 MV photon energy without prosthesis) respectively as shown in Fig. 2. It was observed that there was no significant difference in PTV coverage. Similarly, *D*_2%_ were 49.40 ± 0.84, 49.05 ± 0.76, 48.97 ± 0.91, and 48.57 ± 0.85 Gy for 4FMC6MV, 4FMC6MV_WP, 4FCC6MV, and 4FCC6MV_WP respectively. It was noted that Monte-Carlo algorithm is significantly better for reducing tail of DVH in case of both the cases (*P* = 0.001). *D*_50_ and *D*_mean_ were significantly different for CCC in case of with and without prosthesis, and the results were plotted and shown in Fig. 3. Homogeneity index was found to be more homogenous with Monte-Carlo algorithm (*P* = 0.019 and *P* = 0.001). Other parameters that were evaluated are shown in Table 1.

**Table 1:** DVH analysis of PTV

		1	2	3	4	*P* value			
	Technique/parameter	4FMC6MV	4FMC6MV_WP	4FCC6MV	4FCC6MV_WP	1 vs. 2	3 vs. 4	1 vs.3	2 vs. 4
PTV	*D*_98%_ (in Gy)	44.49 ± 0.11	44.51 ± 0.13	44.39 ± 0.22	44.45 ± 0.16	NS	NS	NS	0.015
	*D*_2_% (in Gy)	49.40 ± 0.84	49.05 ± 0.76	48.97 ± 0.91	48.57 ± 0.85	NS	NS	0.001	0.001
	*D*_50%_ (in Gy)	47.16 ± 0.48	46.85 ± 0.55	47.16 ± 0.52	46.74 ± 0.60	NS	0.02	NS	0.001
	*D*_max_ (in Gy)	52.15 ± 1.28	51.91 ± 0.84	50.14 ± 1.07	49.99 ± 0.91	NS	NS	0.001	0.001
	*D*_min_ (in Gy)	41.61 ± 0.46	41.96 ± 0.52	41.61 ± 0.46	41.76 ± 0.62	NS	NS	NS	0.005
	*D*_mean_ (in Gy)	47.09 ± 0.44	46.83 ± 0.48	47.01 ± 0.46	46.68 ± 0.51	NS	0.038	NS	0.001
	*V*_107%_ (in %)	19.23 ± 14.01	12.73 ± 14.35	14.74 ± 16.09	8.67 ± 15.35	NS	NS	0.004	0.001
	*V*_110%_ (in %)	3.82 ± 6.08	2.53 ± 6.58	3.02 ± 6.26	1.69 ± 5.19	NS	NS	NS	0.041
	CIRTOG	2.03 ± 0.13	1.97 ± 0.11	1.97 ± 0.12	1.91 ± 0.11	NS	NS	0.001	0.001
	CIHT	0.48 ± 0.03	0.51 ± 0.03	0.51 ± 0.03	0.52 ± 0.03	NS	NS	0.001	0.001
	CN	0.47 ± 0.03	0.49 ± 0.03	0.49 ± 0.03	0.51 ± 0.03	NS	NS	0.001	0.001
	HIICRU	0.11 ± 0.02	0.11 ± 0.02	0.11 ± 0.02	0.09 ± 0.01	NS	NS	0.001	0.001
	HI	1.11 ± 0.02	1.11 ± 0.02	1.11 ± 0.02	1.09 ± 0.02	NS	NS	0.019	0.001
	HIRTOG	1.16 ± 0.03	1.15 ± 0.02	1.11 ± 0.02	1.11 ± 0.02	NS	NS	0.001	0.001

1, 4FMC6MV; 2, 4FMC6MV_WP; 3, 4FCC6MV; 4, 4FCC6MV_WP

*WP* Without prosthesis, *NS* Non-significant

### Organs at risk (OARs)

**Fig. 4:**
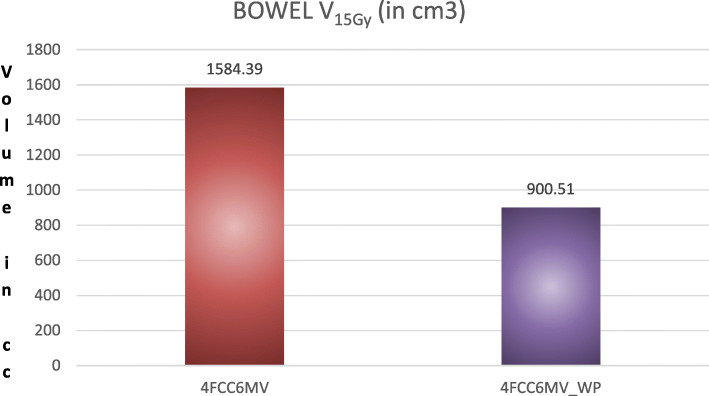
Graphical representation of bowel volume receiving 15 Gy dose

**Fig. 5:**
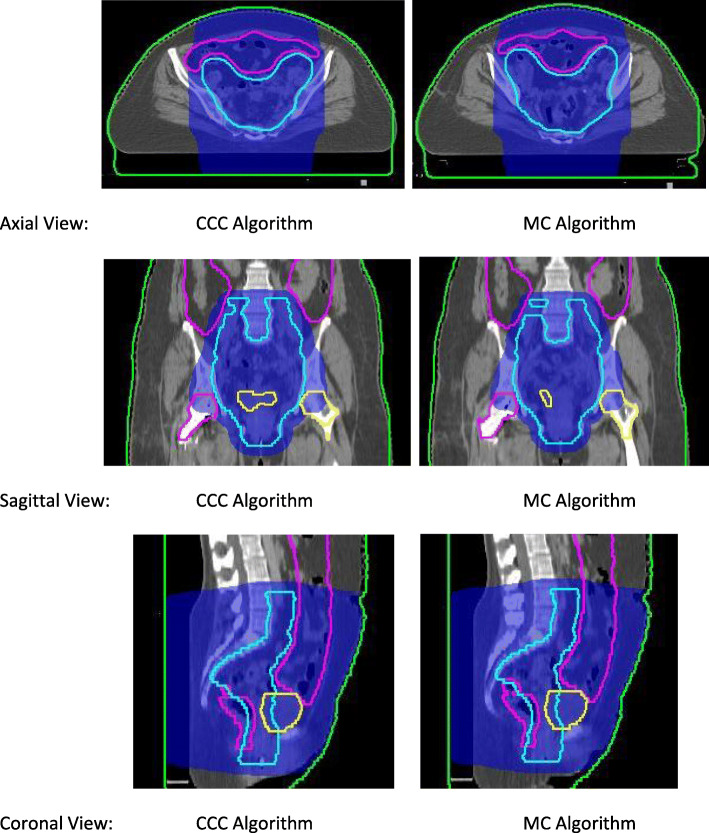
Dose distribution of 20 Gy showing spillage to RFH and prosthesis implant

**Fig. 6:**
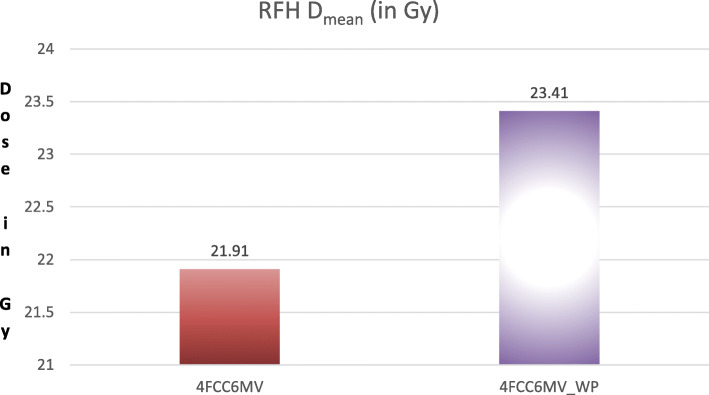
Comparison of mean dose of the right femoral head

Qualified radiation oncologist and medical physicist duo have evaluated the plans and noted the parameters for nearby critical organs as shown in Table 2. It was found that the volume of bowel receiving 15 Gy dose was 1588.97 ± 377.29, 895.89 ± 367.55, 1584.39 ± 367.82, and 900.51 ± 369.89 cm^3^ for 4FMC6MV, 4FMC6MV_WP, 4FCC6MV, and 4FCC6MV_WP respectively. It was observed that there was significant difference among the plans made with and without prosthesis (*P* = 0.015 and 0.022). Graphical representation of *V*_15Gy_ is shown in Fig. 4 for CCC algorithm with and without prosthesis. Similarly, the evaluated mean dose of right femoral head (RFH) was 21.89 ± 3.59, 23.39 ± 2.84, 21.91 ± 3.95, and 23.41 ± 2.89 Gy for 4FMC6MV, 4FMC6MV_WP, 4FCC6MV, and 4FCC6MV_WP respectively as shown in Fig. 5. The significant difference was observed for non-prosthesis cases (*P* = 0.035 and *P* = 0.024). The difference of mean dose calculated with CCC algorithm was shown in Fig. 6.

**Table 2:** Evaluation parameters for OARs and their *P* values

		1	2	3	4	*P* value			
	Technique/parameter	4FMC6MV	4FMC6MV_WP	4FCC6MV	4FCC6MV_WP	1 vs. 2	3 vs. 4	1 vs.3	2 vs. 4
BLADDER	*D*_max_ (in Gy)	50.93 ± 1.21	50.82 ± 1.38	49.13 ± 0.87	49.23 ± 1.19	NS	NS	0.001	0.001
	*D*_mean_ (in Gy)	47.24 ± 0.91	46.61 ± 1.06	46.96 ± 0.96	46.51 ± 1.12	NS	NS	NS	0.036
	*D*_2cc_ (in Gy)	49.42 ± 1.08	49.34 ± 1.17	48.59 ± 0.81	48.72 ± 1.14	NS	NS	0.001	0.001
	*V*_45_ (in %)	94.93 ± 4.19	90.84 ± 8.15	94.37 ± 4.26	90.67 ± 8.42	NS	NS	NS	NS
	*V*_50_ (in %)	1.36 ± 1.92	2.17 ± 7.09	0.03 ± 0.07	1.76 ± 6.34	NS	NS	0.015	NS
RECTUM	*D*_max_ (in Gy)	50.81 ± 1.36	50.52 ± 1.31	48.81 ± 1.27	48.44 ± 1.14	NS	NS	0.001	0.001
	*D*_mean_ (in Gy)	45.48 ± 4.68	45.61 ± 1.47	42.23 ± 9.61	45.22 ± 1.37	NS	NS	NS	0.001
	*D*_2cc_ (in Gy)	48.97 ± 1.41	48.87 ± 1.21	48.36 ± 1.27	47.92 ± 1.15	NS	NS	0.001	0.001
	*V*_45_ (in %)	87.66 ± 15.62	84.92 ± 8.15	75.71 ± 34.28	83.49 ± 8.68	NS	NS	0.012	0.004
	*V*_50_ (in %)	7.99 ± 18.11	4.35 ± 11.32	8.39 ± 21.92	0.31 ± 0.79	NS	NS	NS	NS
BOWEL	*V*_5_ (in %)	82.93 ± 12.44	88.01 ± 15.09	82.59 ± 12.56	87.93 ± 15.11	NS	NS	NS	NS
	*V*_15_ (in %)	63.64 ± 11.95	68.66 ± 15.01	63.64 ± 12.46	69.06 ± 15.01	NS	NS	NS	0.003
	*V*_30_ (in %)	36.37 ± 7.71	36.89 ± 11.01	36.53 ± 7.35	37.17 ± 10.96	NS	NS	NS	0.011
	*V*_15Gy_ (in cm^3^)	1588.97 ± 377.29	895.89 ± 367.55	1584.39 ± 367.82	900.51 ± 369.89	0.015	0.022	NS	0.002
	*V*^45Gy^ (in cm^3^)	157.91 ± 62.54	86.82 ± 66.38	139.84 ± 61.03	73.59 ± 61.53	NS	NS	0.004	0.001
	*D*_mean_ (in Gy)	22.08 ± 3.49	23.26 ± 4.53	22.13 ± 3.51	23.31 ± 4.54	NS	NS	NS	NS
RFH	*D*_mean_ (in Gy)	21.89 ± 3.59	23.39 ± 2.84	21.91 ± 3.95	23.41 ± 2.89	0.035	0.024	NS	NS
LFH	*D*_mean_ (in Gy)	22.37 ± 3.18	23.57 ± 2.76	22.29 ± 3.48	23.58 ± 2.81	NS	NS	NS	NS
TMU		245.21 ± 9.78	243.13 ± 14.68	245.47 ± 8.37	244.41 ± 15.54	NS	NS	NS	NS

1, 4FMC6MV; 2, 4FMC6MV_WP; 3, 4FCC6MV; 4, 4FCC6MV_WP

*WP* Without prosthesis, *RFH* Right femoral head, *LFH* Left femoral head, *TMU* Total monitor unit, *NS* Non-significant

## Discussion

External beam radiation therapy is a treatment of choice in locally advanced cervical malignancies. Advancement in technology has brought in a new era of cancer treatment. As a conventional approach, 2-field technique using antero-posterior and postero-anterior fields was the treatment of choice. But there was unnecessary excessive radiation to the organs, coming in the beam path. It has pushed the planners to use bilateral fields in addition to above portals. This beam arrangement using 4-fields, also known as “box technique,” has set a standard in the field of pelvic cancer treatment.

As per the literature, the box technique was found to be less toxic as compared to the two-field technique [13]. Lateral portals of 4-field technique spares a portion of small bowel superiorly and rectum posteriorly but the same technique with standard portals might be risky without the knowledge of precise tumor volume.

Three-dimensional conformal radiotherapy (3DCRT) facilitates the planner to cover the target with conformal and homogeneous dose, prescribed by the radiation oncologist with volumetric information about the tumor. This technique avoids the geographic miss in terms of conforming the targets. Also, the accuracy of beam calculation algorithms assure the accurate fluence of prescribed dose. There are studies which have shown that Monte-Carlo (MC) can be used as a standard calculation algorithm to compare the dose calculated by other algorithms [14, 15].

The box technique generates the treatment plans with a homogeneous dose distribution and good conformity index in the absence of the prosthesis implant. But the insertion of high-density metallic implant may change the scenario.

The present study has not suggested any major difference between the MC dose calculation algorithm and CCC calculation algorithm in the presence of high-Z metallic implants. Kim et al. [16] also found that pencil-beam convolution algorithm estimated excessive doses for the target than the other two algorithms. The MC algorithm showed better accuracy than the other algorithms. Similarly, Ojala et al. [17] evaluated the accuracy of the Acuros XB (AXB) algorithm in volumetric modulated arc therapy technique by MC simulations. They evaluated the dose distribution using Acuros (AXB) algorithm in comparison with MC simulation technique for clinical situations. In their study, the calculated dose distribution by AXB algorithm was compared to the MC simulation to assess the accuracy of the AXB algorithm in clinical situations. The agreement between AXB algorithm and MC model was very good in vicinity, inside of the implant, and elsewhere. The results verified the accuracy of the AXB algorithm for patient treatment planning with beams traversing through a high-Z material. Paulu et al. [18] has found that the difference between the measured and calculated doses for CCC algorithm was 5–22% and 2–23% for AXB algorithm.

In another study, carried out by Wieslander and Knoos [19] about dose perturbation in the presence of metallic implants (TPS versus MC simulations), two algorithms, i.e., PB and CCC for 6 and 18 MV photon beam, were used. The CCC algorithm showed overall a better agreement with MC simulations than the PB algorithm. Finally, they recommended to use the CCC algorithm to achieve the more accurate dose calculation for both tissues adjacent to the implants and for the planning target volume when the beams are set up to pass through implants.

As per our observations for the delineated critical organs, mean doses with prosthesis implant have shown significant variations for both the algorithms. This may be due to the backscattered dose, caused by the high-density implant. Radiation damage to small bowel tissue can cause chronic or acute radiation-related toxicities such as pain, bloating, nausea, diarrhea, and rectal bleeding which can have a significant impact on patient’s quality of life. Thies et al. [20] reported that toxicity is proportional to the amount of radiation received. Symptoms may occur after 5–12 Gy (low doses) in a fractionated course, but usually occur at higher doses. Volume of bowel receiving 15 Gy dose has shown a significant difference with prosthesis cases. The reason for this might be the dose deposited in the vicinity of implant and added low dose contribution.

A similar pattern was shown for the mean doses of the right femoral head. As we have selected the patients with prosthesis of the right side femoral bone, the portions of the beams entering or exiting the same area might have increased the deposited dose. When a radiation beam passes through prosthesis, changes in the absorbed dose distribution occur due to the increased attenuation of the beams by the prosthesis and interactions at the bone-metal interface. Sung-Lin et al. [21] have also found that none of the planning system can predict the dose accurately in the vicinity or re-buildup in the shadow of the high-density implant. Carolan et al. [22] has highlighted the increase in dose on the distal surface of the prosthesis approximately 35%. The study conducted by Bayatiani et al. [23] also suggested the decrease in dose to the areas which were completely blocked by the metal prosthesis. In addition, the scattering of the beam from the metal surface increases the dose in femoral bone and nearby healthy tissues.

This study investigates that hip prosthesis creates considerable changes in the treatment planning of cervical malignancies like the similar study carried out by Mohammadi et al. [24]. CCC algorithm is comparable with gold standard MC algorithm in terms of tumor coverage and sparing of critical structures except few parameters like volume of small bowel receiving 15 Gy dose and mean dose to femoral head.

## Limitations

The limitation of the study is that the correlation of the present dosimetric data with clinical outcomes needs to be analyzed for better recommendations.

## Conclusion

Four-field box technique is a standard treatment for the treatment of pelvic malignancies. CCC algorithm is in good agreement with MC calculation algorithm in the presence of high-density metallic implants in terms of target coverage and avoidance organ sparing except few parameters. Information regarding density, composition, and orientation of the implant is a useful tool for the planner to apply necessary corrections during treatment planning.

## Data Availability

All data generated or analyzed during this study are included in this published article.

## Abbreviations

CCC: Collapse-cone-convolution
MC: Monte-Carlo
4FMC6MV: Four-field technique calculated with Monte-Carlo algorithm and 6 MV photon energy
4FMC6MV_WP: Four-field technique calculated with Monte-Carlo algorithm and 6 MV photon energy without prosthesis
4FCC6MV: Four-field technique calculated with Collapse-Cone-Convolution algorithm and 6 MV photon energy
4FCC6MV_WP: Four-field technique calculated with Collapse-Cone-Convolution algorithm and 6 MV photon energy without prosthesis
CT: Computed tomography
TPS: Treatment planning system
PBC: Pencil-beam-convolution
PTV: Planning target volume
CTV: Clinical target volume
OAR: Organs at risk
TERMA: Total energy released per unit mass
VEF: Virtual energy fluence
XVMC: Photon voxel Monte-Carlo
TMU: Total monitor units
DVH: Dose-volume histogram
CI: Conformity index
CN: Conformity number
TV_RI_: Target volume covered by the reference isodose
TV: Target volume
*V*_RI_: Volume of reference isodose
RTOG: Radiation Therapy Oncology Group
CI_RTOG_: Conformity index as per RTOG recommendations
CI_HT_: Healthy tissue conformity index
HI: Homogeneity index
HI_ICRU_: Homogeneity index as per ICRU recommendations
HI_RTOG_: Homogeneity index as per RTOG recommendations
*I*_max_: Maximum isodose in the target
RI: Reference isodose
3DCRT: Three-dimensional conformal radiation therapy
AXB: Acuros XB algorithm
